# A Cytosolic Juxtamembrane Interface Modulates Plexin A3 Oligomerization and Signal Transduction

**DOI:** 10.1371/journal.pone.0116368

**Published:** 2015-01-07

**Authors:** Rachael Barton, Danica Palacio, M. Kathryn Iovine, Bryan W. Berger

**Affiliations:** 1 Department of Chemical and Biomolecular Engineering, Lehigh University, Bethlehem, Pennsylvania, United States of America; 2 Department of Biological Sciences, Lehigh University, Bethlehem, Pennsylvania, United States of America; 3 Program in Bioengineering, Lehigh University, Bethlehem, Pennsylvania, United States of America; National Cancer Institute, UNITED STATES

## Abstract

Plexins (plxns) are transmembrane (TM) receptors involved in the guidance of vascular, lymphatic vessel, and neuron growth as well as cancer metastasis. Plxn signaling results in cytosolic GTPase-activating protein activity, and previous research implicates dimerization as important for activation of plxn signaling. Purified, soluble plxn extracellular and cytosolic domains exhibit only weak homomeric interactions, suggesting a role for the plxn TM and juxtamembrane regions in homooligomerization. In this study, we consider a heptad repeat in the *Danio rerio* PlxnA3 cytosolic juxtamembrane domain (JM) for its ability to influence PlxnA3 homooligomerization in TM-domain containing constructs. Site-directed mutagenesis in conjunction with the AraTM assay and bioluminescent energy transfer (BRET²) suggest an interface involving a JM heptad repeat, in particular residue M1281, regulates PlxnA3 homomeric interactions when examined in constructs containing an ectodomain, TM and JM domain. In the presence of a neuropilin-2a co-receptor and semaphorin 3F ligand, disruption to PlxnA3 homodimerization caused by an M1281F mutation is eliminated, suggesting destabilization of the PlxnA3 homodimer in the JM is not sufficient to disrupt co-receptor complex formation. In contrast, enhanced homodimerization of PlxnA3 caused by mutation M1281L remains even in the presence of ligand semaphorin 3F and co-receptor neuropilin-2a. Consistent with this pattern of PlxnA3 dimerization in the presence of ligand and co-receptor, destabilizing mutations to PlxnA3 homodimerization (M1281F) are able to rescue motor patterning defects in *sidetracked* zebrafish embryos, whereas mutations that enhance PlxnA3 homodimerization (M1281L) are not. Collectively, our results indicate the JM heptad repeat, in particular residue M1281, forms a switchable interface that modulates both PlxnA3 homomeric interactions and signal transduction.

## Introduction

Plexins (plxns) are a family of type I transmembrane (TM) receptors involved in neuronal, vascular, and lymphatic development as well as zebrafish fin regeneration in conjunction with semaphorins (semas), their ligand binding partners [1–13]. Class A plxns are known to interact with the secreted class 3 semaphorins, and in this system, neuropilins (nrps) are necessary co-receptors [1–3, 10, 14]. In the plxn-nrp-sema signaling complex, semas serve as the guidance cue, directing the plxn-nrp-expressing cell towards or away from the sema source [1–3, 10, 14–17]. The nrp acts to join sema and plxn, dictating specificity of the sema-plxn association and initiating a signal transduction cascade to alter cell motility [1–3, 8, 10, 14–17]. Furthermore, mutations to plxns have been reported in melanomas as well as lung, breast, pancreatic, and prostate cancers, suggesting their altered signaling may play a role in cancer development [4, 18]. As such, understanding plxn-dependent signaling mechanisms are important both in terms of determining their role in development and disease.

**Figure 1:**
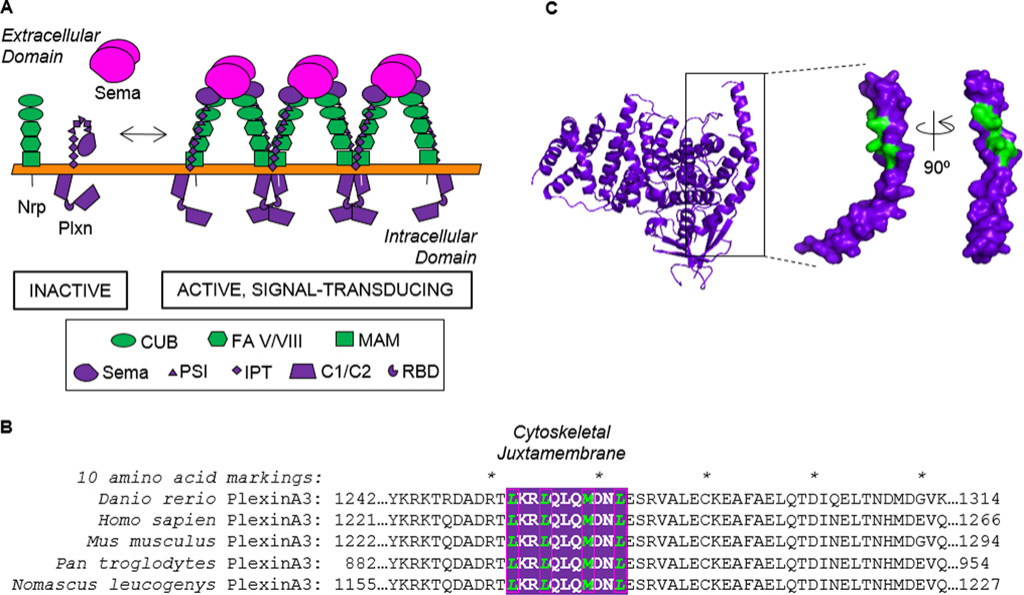
Clustering drives plexin activation. (A) Cartoon illustration indicating the relationship between plexin oligomeric state and function. (B) A cytosolic juxtamembrane heptad repeat in PlxnA3 is conserved across species and may regulate this phenomenon. (C) A crystal structure of the *Mus musculus* PLXNA3 cytosolic domain (PDB # 3IG3) with residues comprising a heptad repeat highlighted in white.

The plxn structure consists of an extracellular sema domain, three plexin-semaphorin-integrin (PSI) domains, and three immunoglobulin, plexin, and transcription factor (IPT) domains, a single-spanning transmembrane domain, and a cytosolic region (CYTO) homologous with Ras GTPase-activating proteins (GAPs) [1, 3]. Deletion studies have shown that the CYTO portion of plxns confers activity provided the TM domain is intact or the CYTO domain is tethered to the membrane and cross-linked in a dimeric or clustered state [19, 20], indicating plxn CYTO oligomerization is important in signal transduction. In particular, overexpression of *Mus musculus* PLXNA1 (mPLXNA1) TM + CYTO in transfected cells is enough to trigger growth cone collapse without the presence of nrp co-receptor or addition of a sema ligand [19]. Similarly, fusion of human PLXNB1 (hPLXNB1) CYTO to the CD2 extracellular + TM domains with the addition of cross-linker also results in cellular contraction [20]. Furthermore, inducing dimerization of the CYTO domains of mPLXNA1, mPLXNA2, mPLXNA4, and mPLXNC1 enhances RapGAP activity over their monomeric counterparts [21]. Overall, these results suggest that plxn CYTO dimerization is important for sema-dependent signal transduction (Fig. 1A).

Interestingly, *in vitro* the CYTO portion of plxns are primarily monomeric, with monomer only detected by analytical ultracentrifugation for mPLXNA3 and hPLXNB1 [6, 7, 9]. Likewise, the full-length mPLXNA2 extracellular domain also exhibits weak homomeric interactions through the extracellular membrane-proximal domains, indicating the extracellular domain may not provide a strong ligand-independent driving force for receptor homodimerization [8]. A dimer of the hPLXNB1 RhoGTPase-binding domain (RBD) has been reported, though this dimer did not form in solution and crystal structures of the full-length hPLXNB1 CYTO domain suggest the contacts between loops responsible for dimerization in the RBD domain alone are replaced by intramolecular interactions [5, 6]. A trimeric structure for the hPLXNB1 CYTO domain has also been reported, though in solution this oligomeric state could not be confirmed, suggesting a high local concentration at the membrane may be necessary for hPLXNB1 CYTO association into dimers and trimers [9]. A recent study involving the hPLXNA1 TM domain demonstrated the TM region has a weak but significant propensity to interact, putatively through alternative forms of specific GxxxG associations that depend on bilayer composition and the presence or absence of the TM domain of the hNRP1 co-receptor [15]. Thus, while in some instances homomeric interactions have been identified for plxns, a unified picture of how plxn homooligomerization occurs is still an area of active research.

In this study, we consider the cytosolic juxtamembrane helix (JM) in the presence of the TM to examine its influence on the homomeric interactions of *Danio rerio* PlxnA3. Using the AraTM assay [22], we examine the TM + JM interactions in cell membranes and identify an interface containing a heptad repeat in the JM as influential to dimerization, with mutations to M1281 in the heptad repeat capable of both enhancing (M1281L) or disrupting (M1281F) PlxnA3 homodimerization. These same interactions are also observed with the full extracellular domain intact with a truncated CYTO domain in a mammalian membrane using the bioluminescence resonance energy transfer (BRET^2^) assay. In the presence of the functionally-relevant Nrp2a co-receptor and SEMA3F ligand [23], we find that disruption to PlxnA3 homomeric interactions via mutation M1281F can be corrected by the addition of a Nrp2a co-receptor and SEMA3F ligand such that PlxnA3 mutant M1281F homodimerizes with a signal similar to that of wild-type (WT) PlxnA3. In contrast, the PlxnA3 mutant M1281L exhibits a greater extent of homodimerization as compared to WT PlxnA3, even in the presence of Nrp2a co-receptor and SEMA3F ligand. To examine the functional effects of these mutations, we injected WT or mutant *plxna3* mRNA into the *sidetracked* (*set*) zebrafish line, which lacks membrane-anchored PlxnA3 and exhibits aberrant motor neuron patterning [24], and examined rescue of WT zebrafish motor neuron patterning. We find that alterations to PlxnA3 homomeric interactions are correlated with phenotype observed: neutral mutations in the presence of a nrp co-receptor and sema ligand (M1281F) rescue zebrafish motor neuron development, whereas mutations that result in enhanced plxn homodimerization in the presence of a nrp co-receptor and sema ligand (M1281L) do not. Collectively, our results suggest the heptad repeat interface in the JM affects PlxnA3 homooligomerization, and residue M1281 in the heptad repeat acts a switch that modulates PlxnA3 oligomeric state and subsequent function.

## Materials and Methods

This study was carried out in strict accordance with the recommendations in the Guide for the Care and Use of Laboratory Animals of the National Institutes of Health. The protocols used for this manuscript were approved by Lehigh’s Institutional Animal Care and Use Committee (IACUC) (approval date 11/8/2013). Lehigh University’s Animal Welfare Assurance Number is A-3877–01. All experiments were performed to minimize pain and discomfort.

### Plasmids

A full-length WT *Danio rerio plxna3* nucleotide template (NCB Accession # AB262187.1, with a translational mutation C1090S) was provided by Dr. Michael Granato (University of Pennsylvania). This construct was subsequently cloned into pcDNA3.1/V5-His-TOPO (Invitrogen) with a C-terminal His-tag as per manufacturer’s instructions. Full-length WT *Danio rerio nrp2a* (NCB Accession # BC162118, Thermo Scientific) was cloned similarly into pcDNA3.1/V5-His-TOPO with a C-terminal FLAG-tag for co-expression studies. The plasmid encoding alkaline phosphatase-tagged (AP-) *SEMA3F* was kindly provided by Dr. Roman J. Giger (University of Michigan).

For the BRET^2^ assay, a truncated coding sequence for WT *plxna3* (amino acids 1–1314 of NCB Accession # BAF81998.1) was cloned into pGFP^2^-N3 (BioSignal Packard) at NheI/HindIII and into pRLuc-N1 (BioSignal Packard) at XhoI/HindIII. For the AraTM assay, the coding sequence for WT *plxna3* TM + JM (amino acids 1241–1314 of NCB Accession # BAF81998.1) was cloned into pAraTM as SacI/KpnI inserts.

PlxnA3 mutants were generated using the QuikChange II Site-Directed Mutagenesis Kit (Agilent Technologies) as per manufacturer’s instructions. For random mutations in pAraTM constructs, EP-PCR was performed as previously described [25].

### AraTM Assay

AraTM measurements were performed as previously described, with the exception that cultures for AraTM measurements were grown for 16–24 hours from glycerol stocks generated from transformed cells grown in selective lysogeny broth (Lennox) medium, rather than from plates. Orientation in the membrane and level of protein expression was confirmed as previously described (S1 Fig.) [22]. Results are reported in terms of the average percent change from WT in slope of green fluorescent protein (GFP) fluorescence *vs*. absorbance determined from a minimum of 11 independent replicates. Standard error was determined by calculating the standard error of these samples and adding the standard error from WT to these values. S2 Fig. illustrates a sample of non-normalized and normalized data for one round of experiments (three replicates) analyzed as previously described [22].

### BRET^2^ Assay

COS-7 cells (ATCC) were grown to 40–90% confluency and transfected at a cell density of 1 x 10^6^ cells/mL in HEPES-buffered saline using 8 µg plasmid DNA per construct and the pre-set COS-7 parameters of the Bio-Rad Gene Pulser XCell. Transfections were then transferred to 2.6 mL media (for BRET^2^ measurements) or 1.5 mL media (for AP-SEMA3F expression). Cultures for BRET^2^ measurements were seeded in eight wells of a 96-well dish (200 µL/well) and cultures for AP-SEMA3F expression consisted of six AP-SEMA3F transfections in a 100 mm dish. Proteins were allowed to grow for two days following the cultivation environment recommended by ATCC with the addition of 1% (v/v) 100x Antibiotic/Antimycotic solution (100 U/mL penicillin G, 100 µg/mL streptomycin, and 0.25 µg/mL amphotericin B) (Hyclone). Following expression, media for BRET^2^ wells receiving AP-SEMA3F treatment was removed and replaced with 200 µL AP-SEMA3F expression media. Treatment occurred for a minimum of 1.5 hours at 37ºC with 5% CO_2_. BRET^2^ measurements proceeded as previously described using a Tecan Infinite F200 multi-well plate reader [26, 27]. Western blots were used to confirm similar expression levels between WT and mutant PlxnA3 BRET^2^ constructs following measurements (EGFP monoclonal antibody, Clontech, and MSX Renilla Luciferase antibody, Millipore). Cell-staining confirmed expression of FLAG-tagged Nrp2a (mouse monoclonal anti-FLAG antibody, Sigma, and Alexa Fluor 546, Life Technologies), and the presence of AP-SEMA3F in the media was confirmed by testing for AP activity via dot blot and a reaction involving 5-bromo 4-chloro 3-indolyl phosphate (Roche) and nitro blue tetrazolium (Roche) previously described [28]. AP-Sema3F binding to Nrp2a- and PlxnA3-transfected cells was confirmed by testing for AP activity on parallel cultures of treated cells as previously described (S3 Fig.) [28].

For analysis, the total luminescence in each well was analyzed, and measurements were only kept for wells with total luminescence values above that of mock-transfected cells. A normalized energy transfer efficiency ratio was determined by calculating the ratio of green luminescence to magenta luminescence in a given well and dividing this value by the ratio of green luminescence to magenta luminescence for mock-transfected cells of that round of experiments. Results presented represent the average normalized energy transfer efficiency ratio determined from a minimum of three independent transfections (a total of at least 21 replicates per condition), with standard error calculated as described for the AraTM assay.

### Zebrafish Housing and Husbandry

Zebrafish are housed in a recirculating system built by Aquatic Habitats (now Pentair). Both 3L tanks (up to 12 fish/tank) and 10 L tanks (up to 30 fish/tank) are used.

The fishroom has a 14:10 light:dark cycle and room temperature varies from 27–29°C (Westerfield, 1993—full ref below). Water quality is automatically monitored and dosed to maintain conductivity (400–600 µS) and pH (6.95–7.30). Nitrogen levels are maintained by a biofilter. A 10% water change occurs daily. Recirculating water is filtered sequentially through pad filters, bag filters, and a carbon canister before circulating over UV lights for sterilization. Fish are fed three times daily, once with brine shrimp (hatched from INVE artemia cysts) and twice with flake food (Aquatox AX5) supplemented with 7.5% micropellets (Hikari), 7.5% golden pearl (300–500 micron, Brine Shrimp direct), and 5% cyclo-peeze (Argent).

Heterozygous *plxna3*/+ parents were set up in breeding cages in the afternoon before gamete collection. In the morning, adult fish were lightly anesthetized with tricane methansulfonate, and embryos were obtained by collecting eggs and sperm separately prior to mixing with water. No animals were sacrificed for this study.

### Zebrafish RNA Injections

The *set* zebrafish line and genotyping protocol were provided by Dr. Michael Granato (University of Pennsylvania) [24, 29]. Capped full-length *plxna3* (WT or mutant) mRNA was generated using the mMESSAGE mMACHINE T7 Transcription Kit (Ambion) according to manufacturer’s instructions using the pcDNA3.1/V5-His-TOPO *plxna3* constructs linearized with XhoI. Embryos from heterozygous or homozygous *set* zebrafish intercrosses were subsequently injected with the *plxna3* mRNA at 1 µg/µL while in the single-cell stage and allowed to grow for 24 hours, at which point chorions were popped and embryos were fixed in 4% paraformaldehyde for 2 hours at room temperature or overnight at 4ºC. Methanol dehydration, collagenase treatment, and embryo staining occurred as previously described with a SV2 antibody (Developmental Studies Hybridoma Bank, University of Iowa) [24]. Embryos were imaged at 20x magnification via fluorescence microscopy (Nikon Eclipse TE2000-U). In previous studies, embryos were rated as positive for the *plxna3*-knockdown (*set*) phenotype if they had two or more branched motor nerves or at least one hemisegment with two or more motor neuron exit points from the spinal cord [30]. In this study, embryos were rated as positive for the *set* phenotype if they exhibited three or more clear ectopic motor neuron exit points. Following imaging, embryos were genotyped with forward primer CCCTTGCAACTGGTGTTTATA and reverse primer AATGTGTCCTTTAGCAGTGG and a subsequent PsiI digestion that cleaved the PCR product generated from *set* DNA.

## Results

### A Juxtamembrane Helix Promotes Oligomerization of PlxnA3

**Figure 2:**
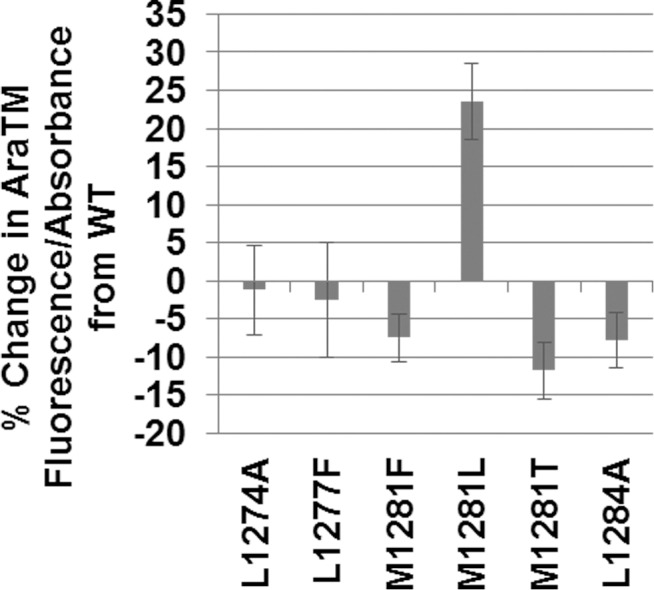
Residues in the PlxnA3 JM heptad repeat promote homomeric interactions in the AraTM assay. Residues on the JM heptad repeat interface influence TM + JM oligomerization, as determined via site-directed mutagenesis. Error bars indicate standard error as determined from a minimum of eleven replicates collected over the course of three experiments.

The PlxnA3 JM domain contains two heptad repeats, which can result in oligomerization of α-helices driven through hydrophobic interactions by hydrophobic residues at the ‘a’ and ‘d’ positions within the heptad repeat [31–33]. Leucine comprises over 30% of interfacial amino acids found in the heptad repeats of α-helical proteins [33]. In the PlxnA3 JM, a heptad repeat occurs with leucines L1274, L1277, and L1284 as well as methionine M1281 (Fig. 1B–C). While methionine is not commonly found in the core of a heptad repeat [33], previous studies have shown that methionine-containing heptad repeats can promote formation of higher-order oligomers rather than dimers in heptad-repeat containing helices [31]. To determine if the heptad repeat in the JM domain influences PlxnA3 homodimerization in the context of the TM-domain-containing receptor, we utilized the AraTM assay. AraTM is a method used to characterize type I integral membrane protein dimerization in *E. coli* membranes, in which the receptor domain of interest is expressed as a fusion to a modified transcriptional activator AraC. If the receptor domains interact, they form a functional AraC dimer that activates transcription of green fluorescent protein (GFP) under control of the *P_BAD_* promoter; hence, the level of GFP expression is directly proportional to dimerization [22]. We expect mutations disrupting the heptad repeat through eliminating the hydrophobic leucine (L1274A, L1284A, L1277F) or introducing polar or larger side-chains instead of methionine (M1281T, M1281F) to disrupt PlxnA3 dimerization, and replacing methionine with the hydrophobic leucine (M1281L) to enhance PlxnA3 dimerization. Consistent with our prediction, we find disruption of the proposed core leucines (M1281F, M1281T and L1284A) significantly disrupts dimerization, whereas introducing an additional leucine into the core (M1281L) enhances dimerization (Fig. 2). Interestingly, disruptive effects are greatest for positions M1281 and L1284 in the heptad repeat, whereas mutations L1274A and L1277F have only a minor effect on dimerization. Collectively, our results indicate the heptad repeat is important for PlxnA3 TM + JM homodimerization, and specific mutations in the second heptad repeat either enhance or diminish dimerization based on their hydrophobicity.

**Figure 3:**
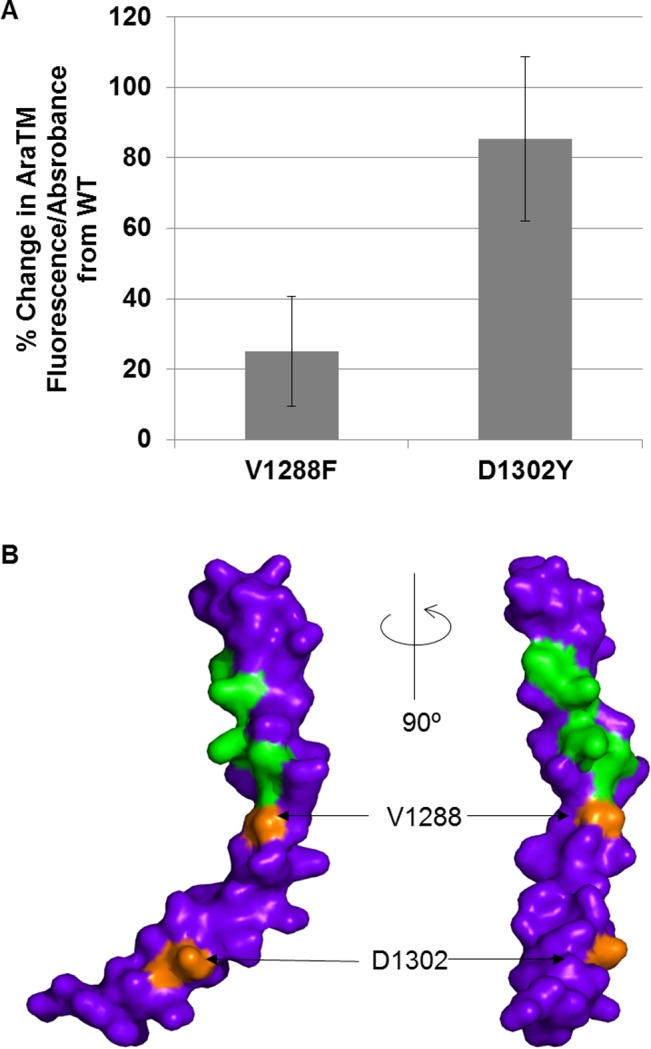
Random mutagenesis yielded additional mutants on the predicted JM interface that alter PlxnA3 homomeric interactions. (A) AraTM results for EP-PCR mutants, where error bars indicate standard error as determined from four replicates. (B) The crystal structure of the *Mus musculus* PLXNA3 cytosolic juxtamembrane domain from PDB # 3IG3 indicates the EP-PCR mutants (gray) lie on the same interface as hydrophobic residues involved in the juxtamembrane heptad repeat (white).

To further identify residues that may impact PlxnA3 TM + JM oligomerization, we performed error-prone PCR (EP-PCR) on the AraTM TM + JM construct and identified either enhancing or disruptive mutants that exhibited significant changes in GFP expression in the assay relative to WT. In particular, two mutants resulted from the EP-PCR studies which consistently enhanced homodimerization relative to wild-type: V1288F and D1302Y (Fig. 3). Based on the mPLXNA3 CYTO crystal structure (PDB # 3IG3) [7], both residues V1288 and D1302 lie along the same face of the JM helix as the heptad repeat (homologous residues mPLXNA3 V1268 and D1282 in PDB # 3IG3). Thus, our unbiased search using EP-PCR identified two JM mutants (V1288F and D1302Y) that also alter homodimerization. Both mutants occur on the same face of the JM helix as the heptad repeat based on the previously reported mPLXNA3 JM crystal structure [7], again consistent with a role for a specific JM interface in regulating PlxnA3 homodimerization.

**Figure 4:**
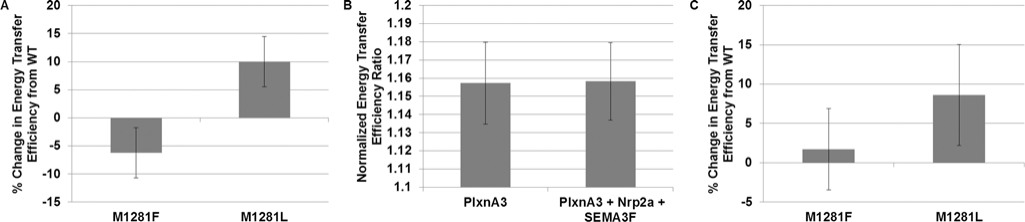
BRET^2^ results indicate alterations to the PlxnA3 JM heptad repeat influence homooligomerization. (A) PlxnA3 homooligomerization in the context of the receptor with the extracellular, TM, and JM domains (residues 1–1314) in a mammalian membrane indicate residues in the JM heptad repeat influence homomeric interactions. (B) The presence of the Nrp2a co-receptor and SEMA3F ligand do not alter homooligomerization of the WT PlxnA3 receptor at the concentrations examined. (C) The presence of the Nrp2a co-receptor and SEMA3F ligand corrects the disruption to PlxnA3 homooligomerization caused by mutation M1281F to that of the WT receptor, whereas mutant M1281L still exhibits significant enhancement to homooligomerization compared to WT. Error bars indicate standard error as determined from a minimum of 21 replicates collected over the course of three experiments.

While we were able to identify potential mutations in the PlxnA3 TM + JM that enhance and disrupt homodimerization, it is important to confirm these effects in the context of the receptor with a full-length extracellular domain in a mammalian membrane. Thus, we introduced mutations identified in the TM + JM region in the AraTM assay and determined their effects on constructs containing the extracellular, TM and JM region (amino acids 1–1314 of NCB Accession # BAF81998.1) using the BRET^2^ assay. We chose to truncate the PlxnA3 JM in the flexible loop region following the α-helix observed in the crystal structure of PlxnA3 CYTO (PDB # 3IG3) to minimize effects of truncations on the secondary structure of the JM region. In these studies, PlxnA3 is co-expressed as an N-terminal fusion to *Renilla* luciferase (RLuc) and modified GFP (GFP^2^). The RLuc-tagged PlxnA3 is capable of converting DeepBlueC to coelenteramide, which emits light at 395 nm; if a GFP^2^-tagged PlxnA3 is nearby or interacting, this light excites the GFP^2^ tag and light is emitted at 510 nm. The ratio of the light emitted at 510 nm to light emitted at 395 nm (the BRET^2^ efficiency ratio) is proportional to the distance between receptors. As such, we would expect mutations disruptive to dimerization to exhibit a lower BRET^2^ efficiency ratio than WT, and those that enhance dimerization to exhibit a higher BRET^2^ efficiency ratio than WT [26, 27]. As shown in Fig. 4A, the JM mutation M1281L enhances PlxnA3 dimerization, while mutation M1281F disrupts it. These results are in agreement with AraTM results for the PlxnA3 TM + JM (Fig. 2), and suggest methionine M1281 acts as a switch within the JM region to regulate PlxnA3 homomeric interactions.

To better understand the role of the JM domains in PlxnA3 oligomerization in the presence of Nrp2 and SEMA3F, which are known interaction partners during neuronal development [23], we performed BRET^2^ measurements on WT and mutant PlxnA3 constructs co-transfected with full-length Nrp2a and treated with media from AP-SEMA3F-expressing cells [34]. The presence of Nrp2a and SEMA3F did not result in significant changes to homodimerization for the WT receptor, as measured by BRET^2^ (Fig. 4B). This suggests that if homomeric interactions of the PlxnA3 JM change upon formation of a Nrp2a-SEMA3F-PlxnA3 co-receptor complex under the investigated experimental conditions, the change does not alter net distance between neighboring PlxnA3 JM domains. However, in the presence of the Nrp2a co-receptor and SEMA3F ligand, mutant M1281F, which is disruptive for homodimerization of PlxnA3 in the absence of Nrp2a and SEMA3F (Fig. 4A), exhibits increased levels of PlxnA3 homodimerization, with a BRET^2^ signal similar to that of WT PlxnA3 (Fig. 4C). Therefore, we conclude co-receptor complex formation promotes PlxnA3 homodimerization independent of disruptive PlxnA3 JM domain mutations such as M1281F. However, mutant M1281L increases PlxnA3 homodimerization independent of whether Nrp2a and SEMA3F are present (Fig. 4A). As such, PlxnA3 homodimerization is influenced by both intrinsic (the heptad repeat within the JM) as well as extrinsic (Nrp2a, SEMA3F) factors, with mutations in the JM region that enhance plxn homodimerization insensitive to the presence of Nrp2a and SEMA3F.

### A Juxtamembrane Heptad Repeat Influences PlxnA3 Function in Zebrafish Motor Neuron Development

**Figure 5:**
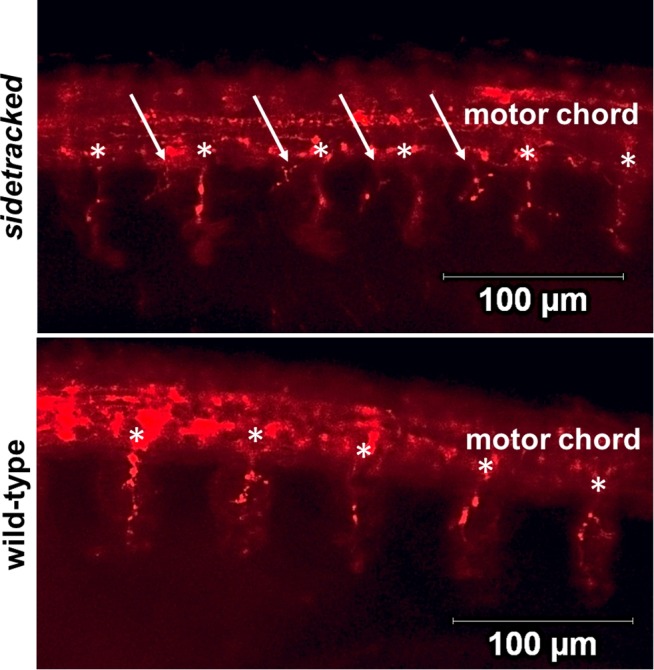
Knockdown of *plxna3* results in aberrant motor neuron patterns. The motor neurons of 24 hour post fertilization *sidetracked* zebrafish embryos (top) exhibit ectopic motor neuron exit points from the motor cord (arrows) compared to WT embryos (bottom). Asterisks indicate endogenous motor neuron exit points. Embryos are oriented anterior (left) to posterior (right).

In zebrafish neuronal development, PlxnA3 acts as a negative guidance receptor. Expression of intact PlxnA3 guides motor axons to exit the spinal cord at midsegments and grow rostrally [24, 30]. The *set* zebrafish line is a transgenic line in which a mutation in the *plxna3* gene results in a truncated form of the receptor incapable of signaling. As such, motor neurons in homozygous *set* mutants exit the spinal cord at ectopic locations and branch (Fig. 5) [24]. To examine if mutations to the PlxnA3 JM affecting homodimerization also affect function, we used the *set* zebrafish line in conjunction with RNA injections of WT or mutant *plxna3* and examined embryos for ectopic exit points [24].

Our results indicate that injection of WT *plxna3* RNA at the single-cell stage of homozygous *set* zebrafish embryos rescues the phenotype of WT zebrafish (i.e. only 36% of *set* embryos injected with WT RNA exhibit ectopic exit points, compared to 80% of uninjected embryos, p<0.005 by Fisher’s Exact Test, FET) (Table 1). Injection of *plxna3* mutant M1281F also rescues the motor neuron patterning of WT zebrafish embryos (i.e. only 33% of M1281F RNA-injected embryos exhibit ectopic exit points, p<0.005 compared to uninjected by FET). In contrast, injection of *plxna3* mutant M1281L RNA, however, failed to rescue motor neuron patterning of the *set* embryos (i.e. 50% of *set* embryos injected with M1281L RNA exhibit ectopic exit points, p = 0.07 compared to uninjected by FET). These results are consistent with PlxnA3 homodimerization results in the presence of the Nrp2a co-receptor and SEMA3F ligand, in which the disruptive mutant M1281F in the absence of Nrp2a-SEMA3F was capable of homodimerization similar to WT in the presence of Nrp2a-SEMA3F, whereas mutant M1281L exhibited a greater extent of homodimerization independent of Nrp2a-SEMA3F (Fig. 4C). Hence, our results suggest an interface containing a heptad repeat in the PlxnA3 cytosolic juxtamembrane, and in particular residue M1281, influences PlxnA3 homooligomerization and subsequent function (Fig. 1). Importantly, our results suggest that promoting PlxnA3 CYTO homomeric interactions, as with mutation M1281L, does not necessarily enhance function, and that a specific conformation within a given oligomeric state is necessary for PlxnA3 function; in this study, the homomeric interactions and oligomeric state formed by the WT and M1281F PlxnA3 are preferred for nrp-plxn-sema signal transduction over the dimeric M1281L PlxnA3 receptor.

**Table 1:** Percentage of Embryos Exhibiting s*idetracked* Phenotype. ^a^

**Type of Injection**	**Number of Embryos Examined**	**Percentage of Embryos Exhibiting s*idetracked* Phenotype**	**P-value Compared to Uninjected**
Uninjected	30	80	1
WT *plxna3* RNA	22	36	0.003
M1281F *plxna3* RNA	12	33	0.009
M1281L *plxna3* RNA	12	50	0.07

^a^P-values were determined using Fisher’s Exact Test.

## Discussion

Previous studies suggest plxn clustering is necessary for activation, though purified extracellular and cytosolic domains exhibit only weak tendencies toward homooligomerization [6, 7, 9, 19–21]. Here, we demonstrate that mutations to the JM heptad repeat interface affect PlxnA3 TM + JM homodimerization (Figs. 2 and 3), with residue M1281 acting as a switch to either enhance (M1281L) or disrupt (M1281F) PlxnA3 homodimerization (Figs. 2 and 4). While the addition of Nrp2a and SEMA3F did not affect WT PlxnA3 homodimerization, disruption of PlxnA3 homodimerization via mutation M1281F was corrected in the presence of both ligand and co-receptor (Fig. 4), and was also able to rescue zebrafish motor neuron patterning in *set* embryos (Fig. 5 and Table 1). In contrast, enhancement of PlxnA3 homodimerization through mutation M1281L was not influenced by the presence of Nrp2a and SEMA3F, and was unable to rescue WT zebrafish motor neuron patterning in *set* embryos (Fig. 5 and Table 1). Thus, M1281 and the associated JM heptad repeat (residues 1281–1287) acts as a switch to regulate the extent of PlxnA3 homodimerization and suggest that interactions promoting weak, but specific, oligomerization of the PlxnA3 JM region are preferred for Nrp2a-PlxnA3-SEMA3F signal transduction versus interactions that enhance PlxnA3 homodimerization at the expense of flexibility in homodimerization during nrp-sema co-receptor formation. In this sense, the JM interface required for PlxnA3 signaling resembles the type of interface required for stabilization of integrin heterodimers, in which a weak, but specific, transmembrane interface was required for switchability between active and inactive states to regulate signal transduction [35]. Given that class A plxns are capable of forming multiple parings with cognate nrp co-receptors and sema ligands (for instance, PLXNA3 participates in NRP1-SEMA3A interactions as well as NRP2-SEMA3F interactions) [3], the additional flexibility in switching between monomer and homooligomeric states for plxns may reflect the necessity of maintaining the flexibility required for proper co-receptor complex formation and subsequent signal transduction.

Previous analyses of the plxn JM region also indicate that switchable interactions involving the heptad repeat region are important for signal transduction. For mPLXNA3, the crystal structure of the cytosolic domain suggests the C-terminal portion of the JM (residues subsequent to L1280 in mPLXNA3, or homologous with residue L1310 in the *Danio rerio* PlxnA3 utilized in this study) makes extensive contacts with the mPLXNA3 GAP domain and mutations to the JM region disrupt function [7]. When including the TM domain of PlxnA3 in the current study, we also find that residues more proximal to the TM influence homodimerization (Figs. 2–4), and in particular mutations at residue M1281 can enhance or disrupt PlxnA3 homodimerization (Figs. 2 and 4). The effects of the M1281 mutation on PlxnA3 function described in this study (Table 1) are also consistent with previous functional analyses of the analogous region in mPLXNA3 and *Drosophila* plxnA (M1261R in mPLXNA3 and M1320R in *Drosophila* plxnA), in which mutants fail to induce growth cone collapse in a PLXNA3-NRP2-SEMA3F growth cone collapse model and exhibit only partial function in a *Drosophila* axon guidance assay [7]. While the effects of arginine mutations on dimerization in these studies is unknown, we do find that mutations which alter the hydrophobicity of the heptad repeat can either enhance (M1281L) or disrupt (M1281F) dimerization, and for M1281L, impair PlxnA3 signaling. Thus, our results indicate a specific oligomeric state and conformation are required for PlxnA3 signal transduction, and the conformation of the activated state requires specific interactions in the heptad repeat of PlxnA3. Conservation of methionine within the heptad repeat of the PlxnA3 JM, and its ability to act as an oligomeric switch, may also reflect its role in creating an oligomeric interface permissive to higher order oligomers versus dimers, thereby providing the flexibility to create a specific interface for signal transduction with switchable interactions.

In summary, our AraTM and BRET^2^ results suggest a specific interface of the PlxnA3 JM domain promotes oligomerization in the context of a membrane-anchored receptor. Mutation to residue M1281 in *Danio rerio* PlxnA3 in particular can enhance or disrupt PlxnA3 homooligomerization, dependent upon type of mutation made (Figs. 2 and 4). Such interaction likely contributes to plxn clustering and subsequent activation (Fig. 1). Our BRET^2^ co-receptor studies suggest Nrp2a and SEMA3F serve to promote PlxnA3 homodimerization for the disruptive mutant M1281F, but not for the dimer-enhancing mutant M1281L. The altered oligomeric state of M1281L in the presence of Nrp2a and SEMA3F correlates with PlxnA3 signaling, in which injection of M1281L mRNA into *set* zebrafish embryos failed to rescue WT motor neuron patterning. As such, residue M1281 regulates PlxnA3 homomeric interactions and subsequent function, suggesting the JM region of plxn forms a specific interface required for signal transduction.

## Supporting Information

S1 Fig Expression and orientation of PlxnA3 TMCY AraTM constructs. (A) Anti-MBP western blot (1:10000 dilution, NEB) of PlxnA3 TMCY AraTM constructs. (B) Maltose complementation test of PlxnA3 TMCY AraTM constructs. (C) Spheroplast assay on the WT PlxnA3 TMCY AraTM construct. Ladder markings are in kDa. The expected molecular weight of PlxnA3 TMCY AraTM constructs is 67 kDa.
(TIF)

S2 Fig AraTM results for a three-replicate round of PlxnA3 TM + JM AraTM measurements. (A) Non-normalized average slope of fluorescence *vs*. absorbance, with error bars indicating standard error determined from three replicates. (B) Average slope of fluorescence *vs*. absorbance represented as a percent change in slope from WT, with error bars indicating standard error of the mutant construct plus standard error of the WT construct. Red bars marked with ‘*’ indicate non-overlapping mean +/- SEM with the WT protein in each graph.
(TIF)

S3 Fig Expression of proteins in the BRET^2^ assay. (A) Anti-GFP (1:1000 dilution, Clontech) western blot confirming expression of PlxnA3-GFP^2^ in the BRET^2^ assay. The expected molecular weights are 176 kDa and 27 kDa for PlxnA3-GFP^2^ and GFP^2^, respectively. (B) Anti-RLuc (1:2500 dilution, Millipore) western blot confirming expression of PlxnA3-RLuc in the BRET^2^ assay. The expected molecular weights are 185 kDa and 36 kDa for PlxnA3-RLuc and RLuc, respectively. (C) Anti-FLAG staining of COS-7 cells expressing FLAG-tagged Nrp2a. No fluorescence was observed in mock-transfected cells. The scale bar in the bottom left frame is the same for all images. (D) Dot blot confirming alkaline phosphatase activity in media from cells transfected with alkaline phosphatase-tagged SEMA3F. (E) Confirmation of alkaline-phosphatase-tagged SEMA3F binding to COS-7 cells expressing Nrp2a and PlxnA3.
(TIF)
