# Supplementary figures and images for: A Cytosolic Juxtamembrane Interface Modulates Plexin A3 Oligomerization and Signal Transduction
Source: PLoS One. 2015 Jan 7;10(1):e0116368. doi: 10.1371/journal.pone.0116368 (PMC4286236; doi:10.1371/journal.pone.0116368)

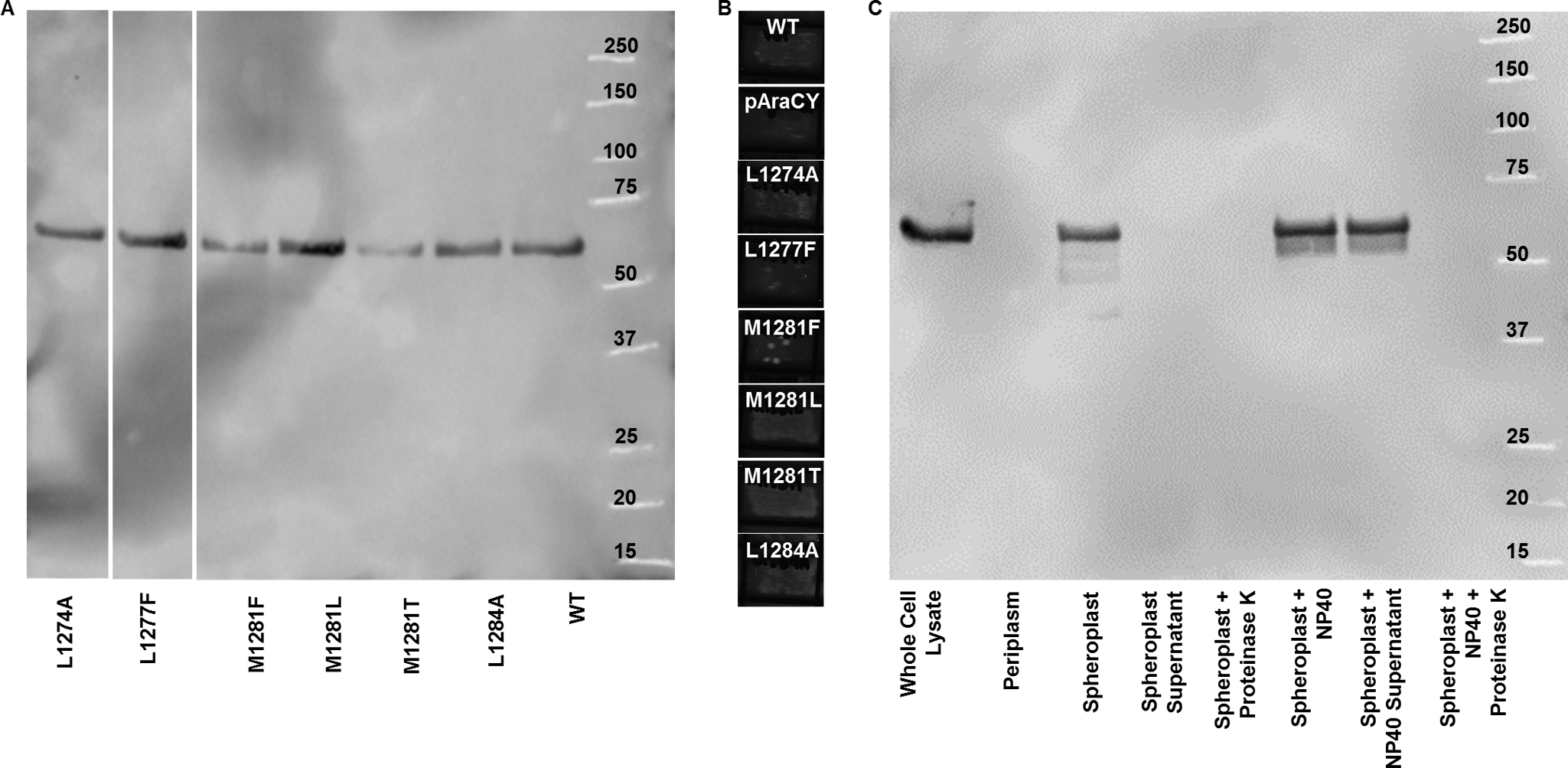

Supplement: S1 Fig — (A) Anti-MBP western blot (1:10000 dilution, NEB) of PlxnA3 TMCY AraTM constructs. (B) Maltose complementation test of PlxnA3 TMCY AraTM constructs. (C) Spheroplast assay on the WT PlxnA3 TMCY AraTM construct. Ladder markings are in kDa. The expected molecular weight of PlxnA3 TMCY AraTM constructs is 67 kDa. (TIF) [file pone.0116368.s001.tif]

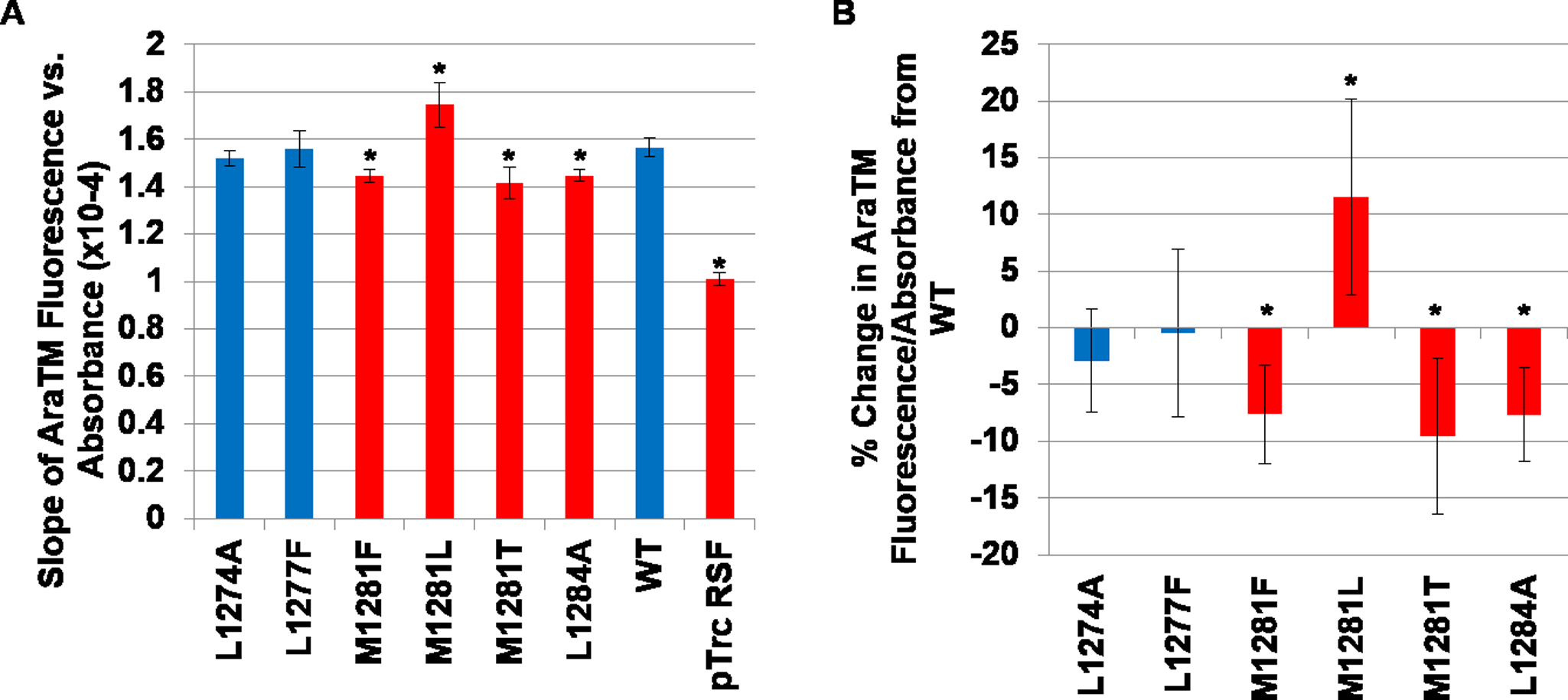

Supplement: S2 Fig — (A) Non-normalized average slope of fluorescence vs. absorbance, with error bars indicating standard error determined from three replicates. (B) Average slope of fluorescence vs. absorbance represented as a percent change in slope from WT, with error bars indicating standard error of the mutant construct plus standard error of the WT construct. Red bars marked with ‘*’ indicate non-overlapping mean +/- SEM with the WT protein in each graph. (TIF) [file pone.0116368.s002.tif]

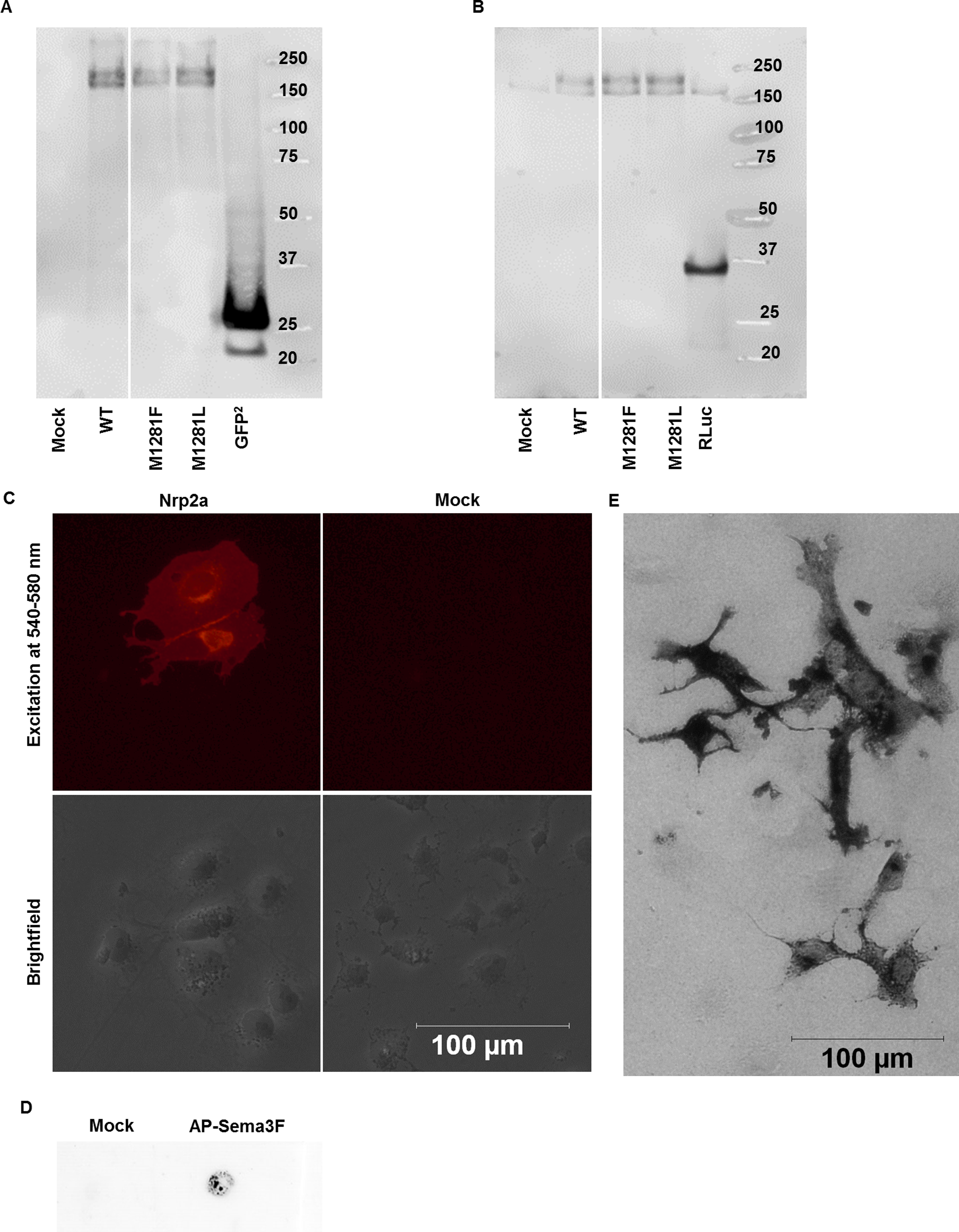

Supplement: S3 Fig — (A) Anti-GFP (1:1000 dilution, Clontech) western blot confirming expression of PlxnA3-GFP2 in the BRET2 assay. The expected molecular weights are 176 kDa and 27 kDa for PlxnA3-GFP2 and GFP2, respectively. (B) Anti-RLuc (1:2500 dilution, Millipore) western blot confirming expression of PlxnA3-RLuc in the BRET2 assay. The expected molecular weights are 185 kDa and 36 kDa for PlxnA3-RLuc and RLuc, respectively. (C) Anti-FLAG staining of COS-7 cells expressing FLAG-tagged Nrp2a. No fluorescence was observed in mock-transfected cells. The scale bar in the bottom left frame is the same for all images. (D) Dot blot confirming alkaline phosphatase activity in media from cells transfected with alkaline phosphatase-tagged SEMA3F. (E) Confirmation of alkaline-phosphatase-tagged SEMA3F binding to COS-7 cells expressing Nrp2a and PlxnA3. (TIF) [file pone.0116368.s003.tif]
